# Large-Scale Mapping of Maize Plant Density Using Multi-Temporal Optical and Radar Data: Models, Potential and Application Strategy

**DOI:** 10.3390/plants14010039

**Published:** 2024-12-26

**Authors:** Jing Xiao, Yuan Zhang, Xin Du, Qiangzi Li, Hongyan Wang, Yueting Wang, Jingyuan Xu, Yong Dong, Yunqi Shen, Sifeng Yan, Shuguang Gong, Haoxuan Hu

**Affiliations:** 1 Aerospace Information Research Institute, Chinese Academy of Sciences, Beijing 100094, China; xiaojing221@mails.ucas.ac.cn (J.X.); duxin@aircas.ac.cn (X.D.); wanghy@aircas.ac.cn (H.W.); wangyueting@aircas.ac.cn (Y.W.); xujingyuan22@mails.ucas.ac.cn (J.X.); dongyong22@mails.ucas.ac.cn (Y.D.); shenyunqi21@mails.ucas.ac.cn (Y.S.); yansifeng23@mails.ucas.ac.cn (S.Y.); gongshuguang23@mails.ucas.ac.cn (S.G.); huhaoxuan23@mails.ucas.ac.cn (H.H.); 2 College of Resources and Environment, University of Chinese Academy of Sciences, Beijing 100049, China

**Keywords:** maize density estimation, mapping strategy, large-scale, multi-temporal

## Abstract

Accurate crop density estimation is critical for effective agricultural resource management, yet existing methods face challenges due to data acquisition difficulties and low model usability caused by inconsistencies between optical and radar imagery. This study presents a novel approach to maize density estimation by integrating optical and radar data, addressing these challenges with a unique mapping strategy. The strategy combines available data selection, key feature extraction, and optimization to improve accuracy across diverse growth stages. By identifying critical features for maize density and incorporating machine learning to explore optimal feature combinations, we developed a multi-temporal model that enhances estimation accuracy, particularly during leaf development, stem elongation, and tasseling stages (R^2^ = 0.602, RMSE = 0.094). Our approach improves performance over single-temporal models, and successful maize density maps were generated for the three typical demonstration counties. This work represents an advancement in large-scale crop density estimation, with the potential to expand to other regions and support precision agriculture efforts, offering a foundation for future research on optimizing agricultural resource management.

## 1. Introduction

Maize is one of the most widely cultivated crops globally, distributed across more than 170 regions, with production highly concentrated in North America, Asia, and South America. It is the third most important cereal crop after wheat and rice. In addition to human food and livestock feed, maize is also used for biofuels and industrial feedstock [1]. Given its versatility and limited cropland availability, maintaining high maize yields is essential to ensure food security [2,3]. Planting density is an important factor affecting both the growth and yield of maize [4,5]. Appropriate planting density optimizes the use of land, water, fertilizer, and light resources, adjusts the distribution of nutrients and the direction of pests and diseases, forms a well-structured plant population, and indirectly improves yield and quality of maize [6,7,8]. Excessive planting density can lead to nutrient deficiencies, increased resource competition, and higher rates of barren stalks and lodging, ultimately reducing yield [9,10]. Conversely, sparse planting wastes land, water, and nutrients, also resulting in lower yield.

Maize density plays an important role in various growth stages of crops and large-scale agricultural decision-making [11]. Using maize density estimation enables the evaluation of sowing quality at the seeding stage, allowing farmers to promptly adjust their planting strategies [12]. During growth, it helps to monitor plant health and optimize field management [13]. At maturity, it aids in yield prediction and the formulation of efficient harvesting [14]. With the rise of modern, mechanized, and intensive farming, large-scale maize planting has become the new normal. As a result, larger-scale retrieving of maize density is essential to help farm managers optimize planting strategies, balancing resource costs, management costs, and yields to boost overall productivity and economic returns [15].

Broadly, the current crop density estimation methods, including maize, mainly include three approaches: (1) ground measurement method, (2) drone imagery estimation method, and (3) satellite imagery estimation method. The ground measurement method relies on manual observations in the field, where researchers or field managers count the number of crop plants and compare it with the study area’s size to determine the density [16]. This type of method is relatively simple, direct, and suitable for use in small-scale experiments or farmland management. The drone imagery estimation method mainly uses drone imagery and intelligent learning algorithms to automatically or semi-automatically identify plants [17,18,19]. These intelligent learning algorithms can be broadly categorized into threshold-based classification methods and deep learning methods. Threshold-based classification methods use pixel thresholds to extract crops, followed by supervised regression or machine learning approaches to estimate the density of crops [20]. For example, Shirzadifar et al. (2020) adopted a K-means clustering algorithm to segment pixels, achieving a field recognition accuracy of 81% [21]. Deep learning methods leverage more advanced architectures for plant identification and density estimation [22,23,24,25,26]. For instance, Liu et al. (2022) proposed a novel deep-learning framework called IntegrateNet, which excels in early-stage maize counting [27]. Similarly, Jin et al. (2017) utilized high-resolution RGB imagery from a drone platform to estimate wheat field plant density with an uncertainty level of just 10% [28]. Although both aforementioned methods are capable of accurately monitoring density, they are unsuitable for the estimation of maize density in primary maize cultivation regions. Ground measurement methods require a considerable input of labor and time when undertaking large-scale estimation. The drone imagery estimation method requires high-resolution images, but acquiring drone images over large-scale areas demands costly equipment and software support.

The satellite imagery estimation method is well-known for its large-scale, synchronous observations with short revisit intervals and high timeliness, providing comprehensive crop density information. It’s primarily used to estimate crop density using satellite image features. Despite its potential, research on using satellite images to estimate crop density is still limited and primarily divided into GIS-based methods and feature parameter methods. The GIS-based method first uses remote sensing images combined with classification methods to identify crops and then uses GIS domain and spatial analysis to estimate crop density across different grid cells [29]. While this method is simple and easy to implement, it tends to have lower accuracy. In contrast, the feature parameter method uses various optical parameters such as Normalized Difference Vegetation Index (NDVI) and Soil Adjusted Vegetation Index (SAVI) obtained from satellites or radar parameters such as Vertical–Vertical Polarization (VV) and Vertical–Horizontal Polarization (VH), combined with machine learning methods to estimate crop density [30]. However, most of these studies are limited to wheat or rice and primarily focus on retrieving data from single growth stages, individual parameter features, and small-scale areas.

At present, there is no established method for the estimation of large-scale maize density in the research literature. Furthermore, while satellite remote sensing provides a basis for large-scale monitoring, reliable methods for high-resolution density estimation over large-scale and multi-temporal stages are still lacking for both maize and other crops. Firstly, there is a lack of methods to integrate satellite images from different sources for estimation purposes. Moreover, due to the non-linear and complex relationship between maize density and other biophysical parameters, the importance of features from different data types of each stage remains unknown which makes it difficult to construct a multi-temporal maize density estimation model. Secondly, the availability of high-resolution satellite images further limits the ability to map large-scale maize density across multiple stages. Effective mapping requires ample optical and radar image features for each stage. However, due to the limitations of weather conditions and image coverage, some subregions are only equipped with optical data, some subregions only provide radar data, and some subregions have both data, which presents a challenge to large-scale mapping.

We have developed a comprehensive system for multi-temporal and large-scale maize density estimation to address the two major challenges in this field. This model integrates optical and radar imagery, covering five key growth stages of maize. It has been designed for maize density estimation in primary cultivation regions, integrating a mapping strategy, thus forming a large-scale maize density mapping. This research aims to advance large-scale, multi-temporal maize density estimation and support broader field management applications.

## 2. Results

### 2.1. Single-Temporal Maize Density Estimation Results

Table 1 summarizes the estimation results for optical, radar, and combined parameters across different growth stages. The estimation accuracy follows the order: Optical + Radar > Radar > Optical. Among the models, Random Forest (RF) and Support Vector Regression (SVR) outperform K-Nearest Neighbors (KNN), likely due to KNN’s sensitivity to noise and its limitations with complex, high-dimensional datasets, whereas RF and SVR better handle such challenges [31].

Maize density estimation performs best during the leaf development (R^2^ = 0.452, RMSE = 0.110) and stem elongation stages (R^2^ = 0.408, RMSE = 0.115). In contrast, accuracy decreases significantly in the middle to late growth stages, such as tasseling, milk ripening, and maturity, where R^2^ drops below 0.250 and RMSE increases above 0.133. This decline is primarily due to increased plant structural complexity and mutual leaf obstruction, which hinder accurate parameter extraction. During the early stages, maize plants have a simpler structure with sparse leaves, facilitating higher estimation accuracy [32,33]. However, as maize develops, the emergence of stems, leaves, and ears, along with intensified competition, complicates spectral reflectance and radar signals, leading to higher error rates [34].

Concerning the different feature parameters, optical parameters alone show poor performance, with R^2^ consistently below 0.155 and occasionally failing to provide retrievable results, highlighting their limited effectiveness for maize density estimation independently. In contrast, radar parameters can explain up to 30% of the variability in maize density during the sowing and stem elongation stages. However, the most effective results are obtained by combining both optical and radar parameters for maize density estimation.

**Figure 1:**
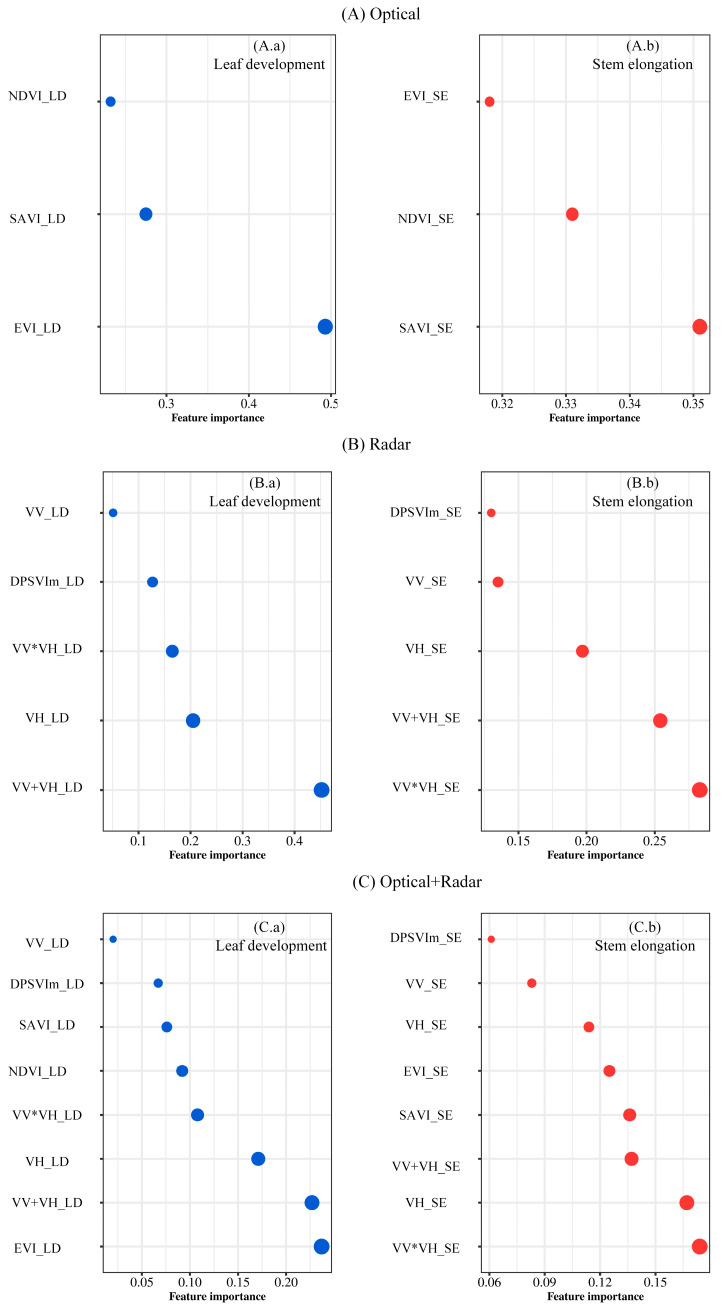
Importance analysis of vegetation parameters for leaf development and stem elongation.

Due to challenges in estimating maize density using optical, radar, or combined parameters during tasseling, milk ripening, and ripening stages, we analyzed the importance of vegetation parameters in RF models during leaf development and stem elongation stages (Figure 1). Results showed a consistent ranking pattern of parameter importance across stages. During leaf development, the Enhanced Vegetation Index (EVI_LD) had the highest explanatory power (score 0.493), followed by SAVI_LD and NDVI_LD. In stem elongation, however, the three optical parameters had similar importance (scores 0.318–0.351). For radar parameters, VV+VH_LD was critical for maize density estimation due to microwave–soil interactions, while VV and the Dual-Pol SAR Vegetation Index modified (DPSVIm) had minimal explanatory power [35]. Among combined parameters, EVI_LD, VV+VH_LD, and VH_LD showed higher importance during leaf development (scores 0.171–0.237), whereas parameter importance was more balanced during stem elongation, with no dominant factor observed.

### 2.2. Multi-Temporal Maize Density Estimation Results

The multi-temporal estimation of maize density demonstrates superior accuracy compared to single-temporal methods (Table 2), with combined optical and radar parameters achieving the highest precision (R^2^ = 0.602, RMSE = 0.094), followed by radar (R^2^ = 0.387, RMSE = 0.116) and optical parameters alone (R^2^ = 0.276, RMSE = 0.127). Among models, RF consistently outperforms SVR and KNN, reaffirming its robustness.

Multi-temporal estimation of maize shows clear advantages, with accuracy surpassing the best single-temporal model performance. The highest accuracy is observed during the leaf development to tasseling phase, highlighting the critical importance of integrating multi-temporal methods for accurate maize density estimation. However, precision declines slightly during later growth stages (e.g., leaf development to milk ripening, R^2^ = 0.566, RMSE = 0.098) due to reduced polarization effects and radar signal attenuation [36]. Moreover, multi-temporal methods also provide a more comprehensive understanding of spatial distribution and growth dynamics, mitigating the limitations of single-temporal approaches.

Combining different types of vegetation parameters shows that optical parameters have relatively poorer performance in estimating maize density across multi-temporal, whereas radar parameters consistently show stable estimation accuracy, achieving an R^2^ of around 0.3. Combined parameters show the best performance, explaining almost 60% of maize density except during the leaf development to stem elongation phases.

The multi-temporal results emphasize the effectiveness of the RF method, which highlights the importance of vegetation parameters during different growth phases: leaf development to stem elongation, tasseling, milk ripening, and maturity. Key parameters such as SAVI_Tasseling, VV*VH_SE, and VV+VH_LD play pivotal roles in maize density estimation. Radar parameters dominate, with maize’s large leaves significantly enhancing radar backscattering and amplifying the sensitivity of these parameters to density variations [37,38]. This dynamic interplay highlights the complex relationship between plant morphology and remote sensing signals.

**Figure 2:**
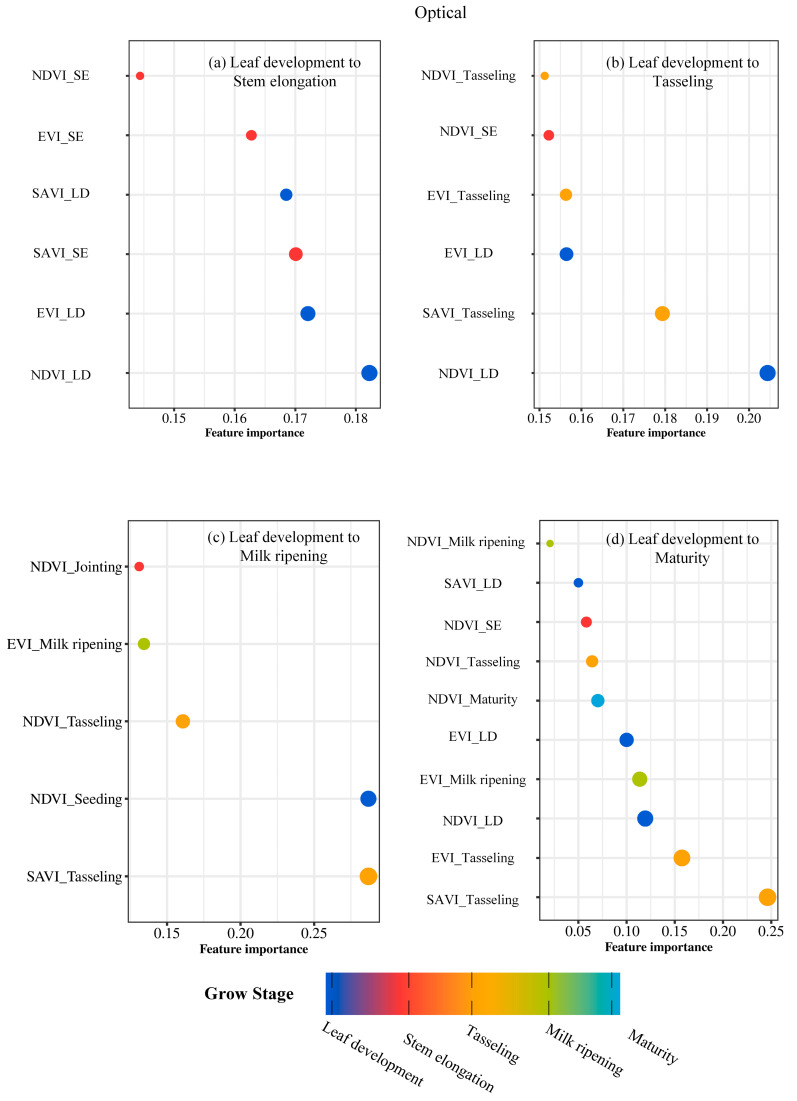
Importance analysis of vegetation parameters across multiple phases in optical.

**Figure 3:**
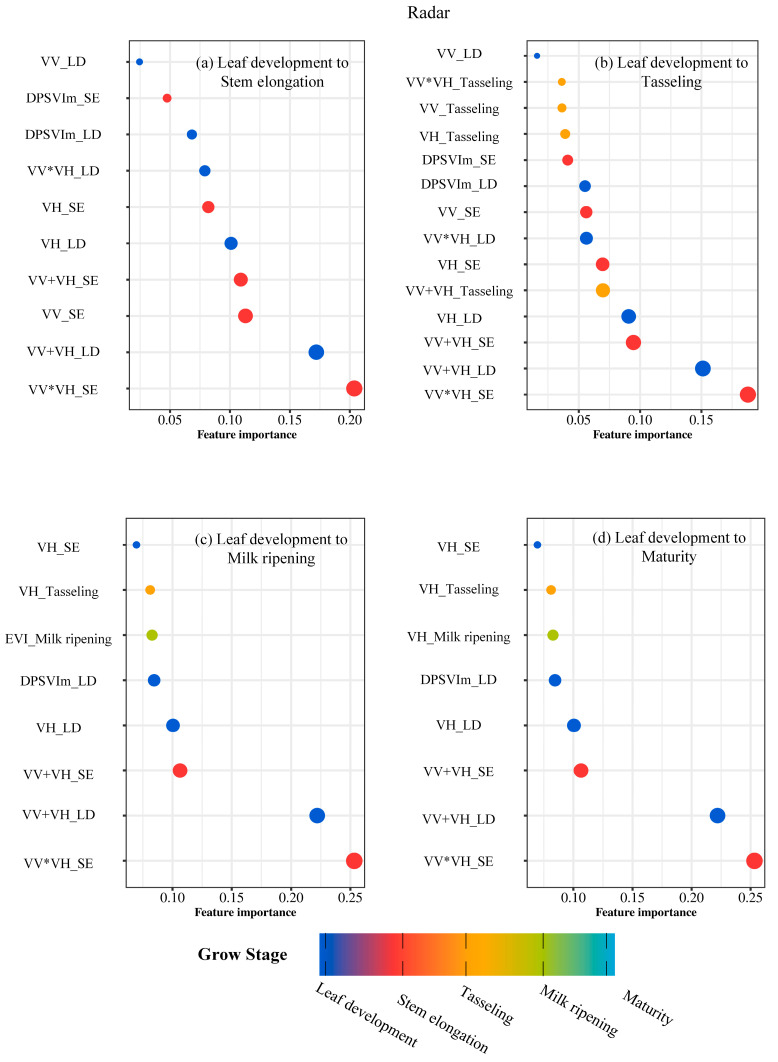
Importance Analysis of Vegetation Parameters Across Multiple Phases in Radar.

**Figure 4:**
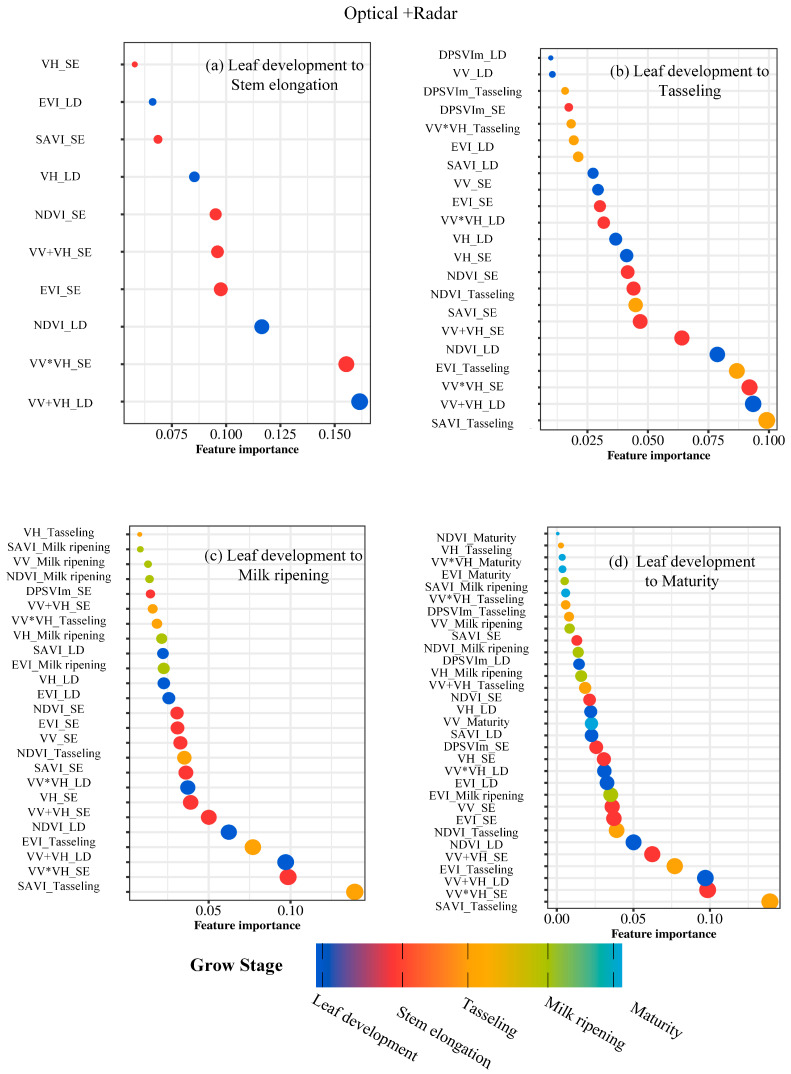
Importance Analysis of Vegetation Parameters Across Multiple Phases in Optical + Radar.

When examining optical parameters individually, SAVI_Tasseling, EVI_Tasseling, and NDVI_LD consistently ranked among the top three in explaining maize density, underscoring the importance of optical parameters during tasseling (Figure 2). For radar parameters, VV*VH_SE, VV+VH_LD, and VV+VH_SE emerged as the most significant, highlighting their key role during the stem elongation stage (Figure 3). Combined estimation results showed a notable improvement in maize density prediction from leaf development to stem elongation and tasseling (Figure 4). The critical importance of SAVI_Tasseling in combined estimation reflects its ability to integrate soil and vegetation reflectance while mitigating soil brightness effects, allowing it to better capture vegetation growth dynamics during tasseling [39].

### 2.3. Mapping Strategy of Large-Scale Maize Density

The maize density mapping strategy is shown in Table 3. The table outlines mapping strategies for maize density, encompassing both single-temporal and multi-temporal estimation methods. Each approach indicates the parameters used, the optimal accuracy achieved, and the models used. Of these, radar data for tasseling, optical data for milk ripening, and optical and combined data for maturity were not shown due to insufficient potential for accurate maize density estimation. From the table we can easily access maize density estimation levels for any grow stage, taking into account both data availability and time constraints in different regions. In cases where data is limited, maize density estimation can be performed using either optical or radar data alone. For urgent needs, consulting the table and prioritizing earlier growth stages can effectively meet maize density estimation requirements.

### 2.4. Case Study on Implementing Maize Density Mapping Strategy

**Figure 5:**
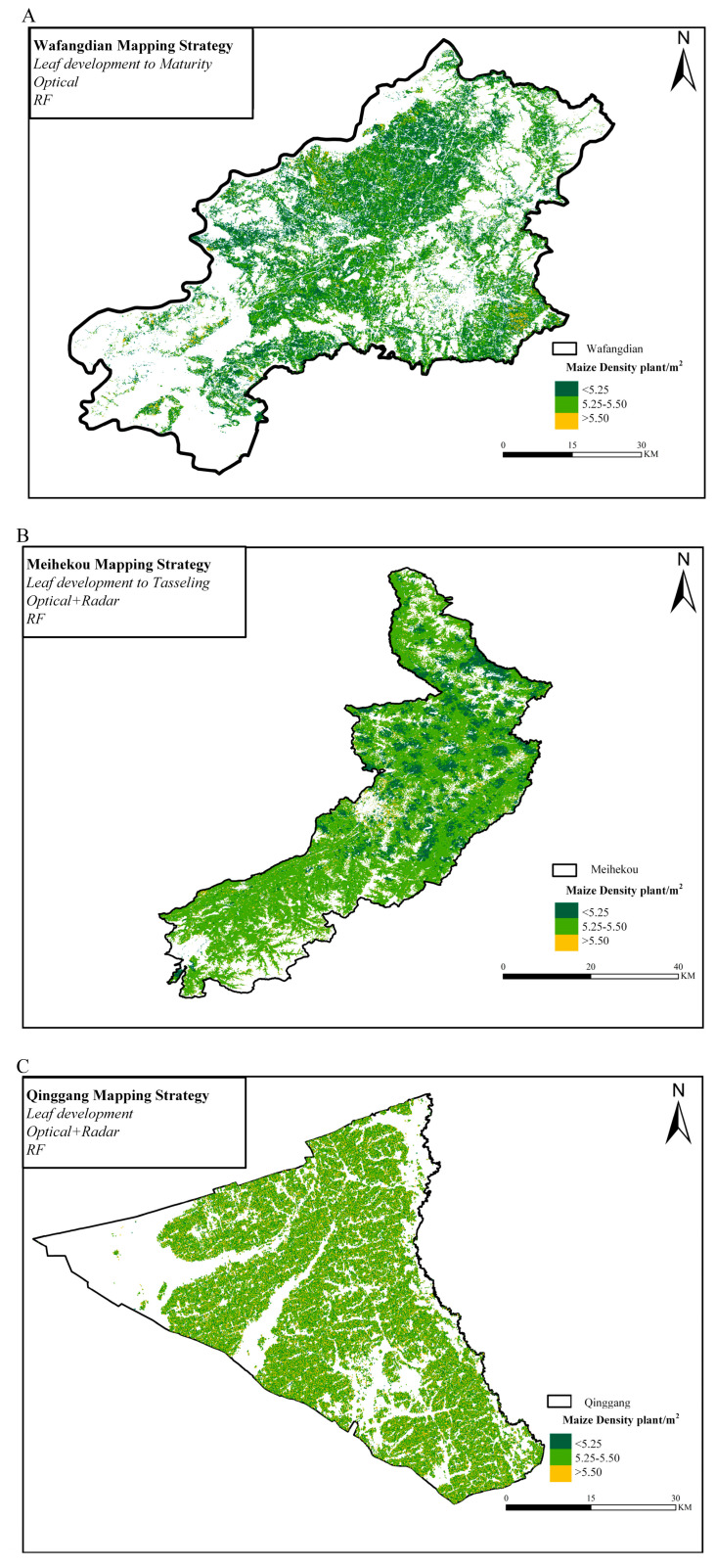
Estimation results of maize density in three typical demonstration counties. (**A**) wafangdian, (**B**) Meihekou, and (**C**) Qinggang.

The mapping strategy W_SM_O_RF is employed to visualize maize density in Wafangdian, M_ST_OR_RF in Meihekou, and Q_S_OR_RF in Qinggang. The resulting maize density maps are presented in Figure 5. The derived data reveals a lower maize density in Wafangdian contrasted with a higher density in Qinggang, with the density across the three typical demonstration counties predominantly ranging from 5.25 to 5.50 plants/m^2^, illustrating the spatial heterogeneity in maize distribution. The estimation differences in maize density across Wafangdian, Meihekou, and Qinggang are driven by a combination of regional agricultural practices, environmental conditions, topographic features, and the specific mapping strategies employed. Wafangdian’s lower maize density may be attributed to less intensive farming and heterogeneous landscapes, while Meihekou, with its moderate maize density [40]. In contrast, Qinggang’s higher density reflects favorable environmental conditions, flat terrain, and improved management practices [41].

## 3. Discussion

### 3.1. Maize Density Estimation Accuracy

In this paper, we developed a large-scale, multi-temporal maize density estimation model by integrating optical features (NDVI, SAVI, EVI) and radar features (VV, VH, VV+VH, VV*VH, DPSVI_m_) with machine learning methods, achieving a maximum accuracy of R^2^ = 0.602 and RMSE = 0.094. Additionally, we constructed a mapping strategy to apply the model for maize density estimation in three typical demonstration counties. In contrast to traditional GIS methods, which are limited to estimating crop density within predefined buffer zones and cannot perform pixel-based density, the approach provides a more accurate and detailed analysis of maize density [29]. Furthermore, existing research on remote sensing for crop density estimation, including maize, typically relies either on optical data or radar data alone for density estimation. In terms of performance, single vegetation index regression models exhibit lower accuracy, with prediction errors exceeding 20% [42]. Our results outperform most remote sensing estimation results with lower prediction errors and are even comparable to some drone-based inversion results [30,43]. Such large-scale methods are limited in accurately retrieving maize density due to the constraints of using single optical or radar features. In contrast, small-scale crop density capturing, which combines high-resolution drone imagery with deep learning algorithms, achieves high accuracy with R^2^ values above 0.8. However, this accuracy is only applicable to the leaf development stage and does not support crop density retrieving in later growth stages or large-scale areas [25,26].

In addition to the aforementioned accuracy advantages, the analysis of feature importance has revealed the effectiveness of the VV+VH, VV*VH, and SAVI features in maize density estimation. Previous research has demonstrated a robust correlation between optical features associated with vegetation cover, such as SAVI, and crop density [44,45]. Furthermore, research suggests that combining VV and VH enhances the estimation accuracy of biochemical parameters, including crop density, throughout the maize growing cycle [46]. Our findings also substantiate these conclusions.

### 3.2. Advantages of the Estimation Framework

In this study, we introduce an innovative model for large-scale, multi-temporal maize density estimation, combined with the mapping strategy to achieve flexible and large-scale maize density mapping. This advancement addresses the gap in large-scale maize density estimation and offers technical support for precision agriculture. Our research has the following advantages:

Firstly, the model addresses the challenge of large-scale maize density estimation. Previous methods, such as ground surveys or localized drone monitoring, are constrained by temporal and spatial limitations, making them unsuitable for large-scale monitoring [19,26]. In contrast, our approach employs the continuous monitoring capabilities of sentinel data, facilitating comprehensive and systematic observation of maize over large-scale areas [47,48]. By combining sentinel data with the multi-temporal maize density estimation model, we can perform maize density estimation at local or regional scales and extend this capability to a global level, enabling comprehensive global maize density retrieving.

Secondly, we have developed a multi-temporal model to enable large-scale maize density estimation. This model integrates data from the five key maize growth stages, from leaf development to maturity, with multiple sentinel images, forming a comprehensive model that encompasses multiple stages and data sources. Although maize density itself is relatively stable, the findings indicate that using optical and radar parameters from pivotal growth stages to encompass both surface and internal maize structures enables a more comprehensive estimation of the factors influencing image-based maize density, which improves the accuracy of the estimation. Moreover, the model offers both a single-temporal model and a multi-temporal model, providing a flexible framework for maize density estimation. The single-temporal model focuses on maize density estimation during a single growth stage, while the multi-temporal model integrates features from multiple growth stages, further enhancing the accuracy of maize density estimation. Our multi-temporal model design not only meets the needs of diverse scenarios but also adapts to different regions and growing stages for maize density retrieving.

Finally, we developed a mapping strategy combined with the large-scale, multi-temporal model for large-scale maize density mapping. This strategy considers not only the maize growth cycle and the availability of imagery but also time constraints. By adopting the mapping strategy, tailoring the estimation for each sub-region based on the available optical and radar data to maximize the value of existing data and ensure reliable maize density estimation, even in areas with limited data. Meanwhile, in regions where timely updates of maize density are critical due to government or other institutional requirements, the strategy prioritizes time over accuracy. This ensures that maize density maps are generated rapidly. In summary, the strategy is flexible, addressing both data availability and time constraints. By integrating mapping strategies with the multi-temporal model, we are able to map large-scale maize density efficiently.

### 3.3. Potential Applications

Maize density plays a critical role in agricultural production [11]. Optimizing planting density can maximize the use of land resources, leading to significant improvements in both maize yield and quality [49]. Extensive production experience has demonstrated that adjusting planting density is an effective and economical strategy for boosting yields [50,51]. By using the large-scale, multi-temporal maize density estimation model and mapping strategy, we can quickly map maize density across different areas and growth stages. This ability is essential for optimizing agricultural resource management.

First, large-scale maize density data is essential for developing a planting strategy. By retrieving large-scale maize density, we can identify optimal planting density patterns for different regions and offer tailored recommendations based on local conditions, thereby enhancing yield and economic returns [15]. For example, analyzing yield differences across different regions with varying planting densities helps local farmers choose suitable maize densities according to soil conditions, water availability, and climate characteristics. Furthermore, maize density is vital for the development of crop models. Large-scale data captures a wider array of maize density conditions, enabling more accurate predictions of crop growth parameters (such as LAI and chlorophyll content) and yield potential, which supports precision agriculture [36,52]. Additionally, monitoring trends in maize density over multiple years can help assess the impacts of climate change and technological advancements on crops, guiding future agricultural planning.

Second, multi-temporal maize density provides flexible growth information for maize. By retrieving maize density at different growth stages, abnormal growth conditions can be detected in time, allowing for the adoption of appropriate management. For example, low density might lead to insufficient space for later growth, while overly dense planting can suppress individual plant development and reduce yield [4,5]. Regular retrieving maize density enables agricultural producers to adjust irrigation, fertilization, and other management practices as needed, ensuring optimal maize health and maximizing yield [53,54].

### 3.4. Limitations and Possible Improvements

To meet the demands of large-scale mapping, the sampling process is both time-consuming and labor-intensive, limiting the amount of sample data available. Future improvements could focus on developing methods to augment limited maize density without altering its distribution, thereby increasing the sample size and improving the model’s ability to generalize across different maize density conditions. With a larger dataset, we can employ deep learning algorithms, such as Convolutional Neural Networks, to automatically extract complex features and learn patterns from various stages of the maize growth cycle, thereby enhancing the accuracy of maize density estimation.

## 4. Materials and Methods

**Figure 6:**
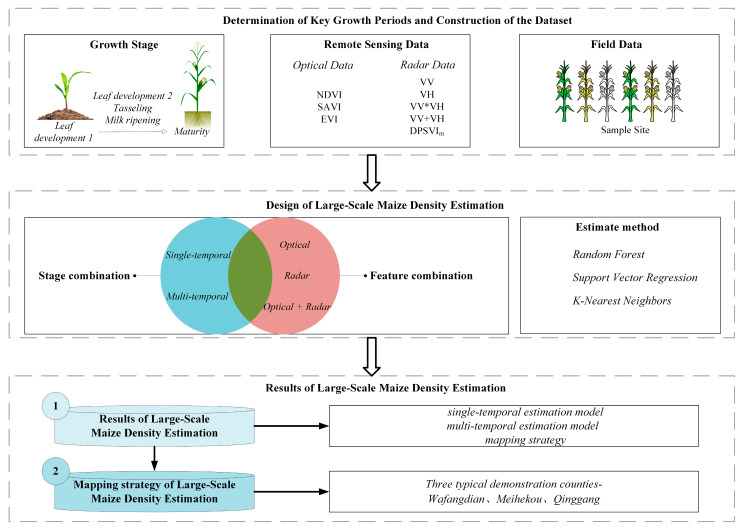
Technical flowchart for maize density estimation.

The study explores the use of various feature sets of key growth stages retrieved from optical and radar remote sensing data for large-scale and multi-temporal maize density mapping. Various machine learning models, including Random Forest (RF), K-Nearest Neighbors (KNN), and Support Vector Regression (SVR), were employed to develop the maize plant density estimation model. The significance of each feature and growth stage in the estimation process was assessed, highlighting the practical applicability of both optical and radar imagery. The flowchart for maize density estimation through optical and radar temporal data is shown in Figure 6.

### 4.1. Study Area

**Figure 7:**
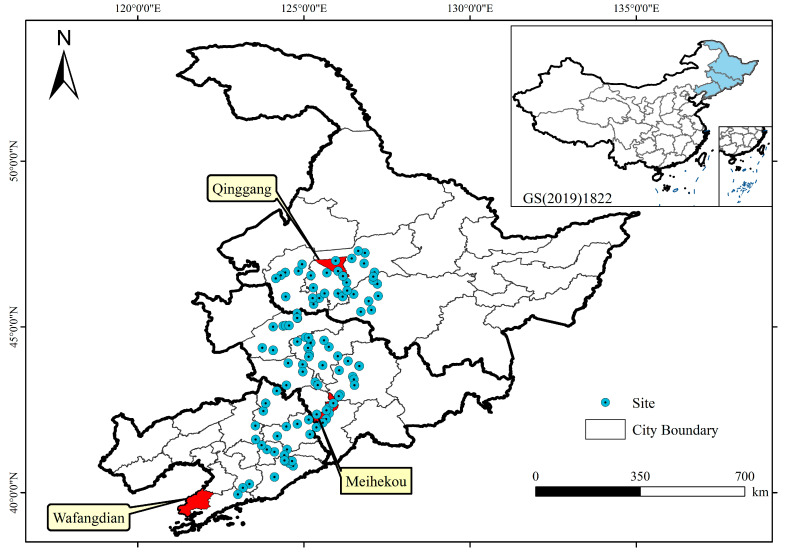
Location of the study area and three typical demonstration counties.

The study encompasses Heilongjiang, Jilin, and Liaoning provinces in China (48° N–55° N, 118° E–135° E), situated within a temperate semi-humid continental monsoon climate (Figure 7.). The region’s thermal resources are modest, with topography that is predominantly flat and mountainous. The average annual temperature registers at 2 °C, and the mean annual precipitation is approximately 500 mm. This area stands as China’s preeminent grain cultivation and export nexus, serving as a linchpin for the food security [55]. Maize is the primary agricultural staple, occupying 60% of the grain crop sowing area and contributing to 34% of the nation’s annual maize yield. The agricultural regimen here is characterized by a single cropping cycle per annum [56].

Three typical demonstration counties, Qinggang in Heilongjiang Province, Meihekou in Jilin Province, and Wafangdian in Liaoning Province, were selected within the study area to illustrate the implementation of the large-scale maize density mapping strategy. The three counties were selected to represent different combinations of factors such as maize densities, image coverage, and agricultural management practices (see the Section 4.7 for detailed information).

### 4.2. Data Sources and Preprocessing

#### 4.2.1. Field Survey Data

Two field trips were conducted in June and September 2023 to collect maize densities. The maize density was measured at a total of 102 field sites, with the first sampling trip encompassing 40 sites and the subsequent trip covering the remaining 62 sites. Each site spanned an area of at least 1 km by 1 km. To ensure comprehensive coverage of field variability, three plots were demarcated at each site, spaced over 10 m apart [57]. To accurately gauge the maize density per plot, a measuring tape of at least 5 m in length and width is employed to tally the number of maize plants. The resulting count, in conjunction with the tape’s measurements, facilitates the computation of the plot’s maize density. An aggregate maize density for each site is established by averaging the densities from three individual plots, thereby yielding an extensive array of 102 distinct maize density data points.

#### 4.2.2. Remote Sensing Data

The aim is to develop comprehensive image features encompassing various types and periods, utilizing high-resolution optical and radar data to facilitate the estimation of maize density on a large-scale. In this study, we selected Sentinel-2A and Sentinel-1 GRD images, which are well-suited for large-scale maize density estimation due to their accessibility, extensive global coverage, and precise data alignment capabilities.

The Level-2A product, derived from Sentinel-2 imagery accessed via the Google Earth Engine (https://earthengine.google.com/, accessed on 10 October 2023), represents an advancement from the Level-1C baseline product, having been subjected to atmospheric correction processes. This refinement transforms the apparent atmospheric reflectance into an accurate surface reflectance data [58]. Averaging the remote sensing data from three plot points provided insights for site points. Henceforth, the discussion will shift from plot-centric to site-centric to enhance clarity and precision.

The GRD data underwent preprocessing in GEE, including thermal noise removal, radiometric calibration, and terrain correction, to produce orthorectified products [59]. To mitigate discrepancies due to maize growth variability within the maize leaf development to maturity cycle, the GRD data were constrained to a seven-day window relative to the Sentinel-2 imagery.

Sentinel-2A and Sentinel-1 GRD data for 2023 were obtained for both sites and three typical demonstration counties. The images from sites were used for modeling, while those from the typical demonstration counties were used to illustrate different mapping strategies. Data for sites were acquired throughout the maize growth cycle to fulfill the needs of multi-temporal maize density modeling. Different types of data were obtained for the three typical demonstration counties at different times due to varying estimation strategies. Optical and radar images during the leaf development stage were obtained for Qinggang, optical and radar images from leaf development to tasseling growth stage were obtained for Meihekou, and optical images from leaf development to the maturity stage were obtained for Wafangdian. All data were acquired with minimal cloud cover guaranteed during acquisition.

### 4.3. Growth Stage Division

Drawing from the FAO’s recommended growth stages, the BBCH scale, scholarly research, historical planting data of maize in the northeastern provinces, and the region’s actual conditions, the maize growth cycle in the northeastern provinces is identified as encompassing six primary stages from mid-May to early September [55,60,61,62]. These stages include sowing, leaf development, stem elongation, tasseling, milk ripening, and maturity, with each stage lasting approximately 20 days (Table 4). Given that the crops are not yet visible above ground during the sowing stage, this study focuses on the five growth stages of maize from leaf development to maturity [63]. Based on the delineation of these growth stages, optical and radar images corresponding to each stage were selected for each site.

### 4.4. Optical and Radar Feature Selection and Calculation

From an optical perspective, the ability to characterize maize density is directly linked to vegetation indices. These indices enhance vegetation features by combining or enhancing spectral bands in optical images while minimizing soil and atmospheric influences, thereby indirectly reflecting variations in maize density [64,65,66]. From a radar perspective, polarization information can penetrate the maize canopy, generating multiple echo signals that permit the acquisition of data about varying degrees of maize density [67,68].

Based on the findings of the literature review, a set of refined optical and radar features was selected for the estimation of maize density, encompassing three optical parameters and five polarization indices (Table 5). The optical parameters include NDVI, SAVI, and EVI, while the polarization indices comprise VV, VH, VV+VH, VV*VH, and DPSVI_m_. These parameters were computed for each of the five growth stages considered (leaf development to maturity).

### 4.5. Estimation Models

#### 4.5.1. Random Forest

The Random Forest (RF) algorithm, a widely utilized ensemble learning method stemming from decision trees, is non-parametric and adept at generating unbiased error estimates internally without necessitating dimensionality reduction [74]. Its proficiency in handling high-dimensional, nonlinear remote sensing data makes it particularly suitable for such analyses. Given its capacity to process inputs with complex feature sets, robustness against noise, resistance to overfitting, and ability to assign importance scores to different vegetation parameters during regression, this study employs the random forest model to assess maize density at each site [75,76]. The RF model is implemented in Python 3.9.2 utilizing the RandomForestRegressor from the sklearn.ensemble module. For each iteration of the modeling process, random_state is fixed at 42 to ensure reproducibility. Optimal hyperparameters such as n_estimators (the count of decision trees), max_depth (the maximum tree depth), min_samples_split (the minimum node split count), and min_samples_leaf (the minimum count of leaf nodes) are determined through five-fold cross-validation. This technique enhances the model’s ability to generalize and mitigates the risk of overfitting. The importance of various vegetation parameters is assessed using a Gini impurity-based method, which is known for its fast-training speed and insensitivity to outliers and noisy data [77].

#### 4.5.2. Support Vector Regression

Support Vector Regression (SVR), predicated on the robust statistical framework of support vector machines, excels in modeling nonlinear interdependencies within multidimensional feature spaces [78]. The quintessence of SVR lies in its capacity to pinpoint a hyperplane that distinctly delineates the nexus between input predictors and target outputs. The judicious selection of the kernel function is paramount, with the Radial Basis Function (RBF) kernel being empirically validated as preeminent for prognostication and estimation tasks in the agronomics [79,80]. This inquiry harnesses the RBF kernel to deduce and quantify maize density. The SVR model is operationalized via the ‘SVR’ module housed within the ‘sklearn.svm’ suite in Python.

#### 4.5.3. K-Nearest Neighbors

K-Nearest Neighbors (KNN) algorithm predicates its predictions on the premise of similarity, leveraging spatial proximity to infer the values of the target variable [31]. As a non-parametric learning approach, it exhibits formidable resilience against outliers and noise, rendering it versatile across diverse data modalities. The algorithm’s pivotal parameter, the number of neighbors (K), inversely correlates with model complexity; a diminutive K augments sensitivity to noise, whereas an amplified K enhances noise immunity [81]. In the investigation, the KNN model was instantiated in Python utilizing the ‘KNeighborsRegressor’ from the ‘sklearn.neighbors’ module, with empirical evaluations culminating in the optimal selection of K as 5.

### 4.6. Experimental Design for Maize Density Estimation

To achieve estimation research on large-scale maize density, an estimation method was designed that encompasses multiple growth stages and multiple parameter types. By identifying the critical growth phase of maize and developing a set of optical radar parameters that are correlated with maize density, three machine learning methods are integrated to estimate maize density. To ensure accurate and efficient estimation of maize density across different stages, two approaches have been implemented: single-temporal and multi-temporal estimation. The single-temporal aims to explore how remote sensing information from different stages represents maize density, while the multi-temporal integrates the capability of various vegetation parameters across multiple growth stages to comprehensively characterize maize density.

#### 4.6.1. Single-Temporal Maize Density Estimation

In the study, we refined the maize density estimation model by integrating single-temporal stage vegetation parameters. The precision of maize density estimation was assessed using distinct optical and radar features, as well as their combined application across various phenological stages. This approach identified the most effective single-temporal feature set for maize density estimation and established a hierarchy of importance among the vegetation parameters. Utilizing RF, SVR, and KNN algorithms, we processed both individual and combined optical and radar features corresponding to key developmental stages—leaf development, stem elongation, tasseling, milk ripening, and maturity—to accurately estimate maize density at each stage (Table 6).

#### 4.6.2. Multi-Temporal Maize Density Estimation

In multi-temporal maize density estimation, we sought to determine the optimal composite period for maize density estimation, establish a ranking of vegetation parameter importance, and assess the accuracy of maize density estimation. Four types of multi-temporal maize density estimation were conducted, corresponding to the characteristics of maize’s five key growth stages (Table 7). For each phase, we constructed maize density estimation models using optical parameters, radar parameters, and their integration, with specific parameter features delineated in Table 6.

#### 4.6.3. Accuracy Evaluation and Optimal Model Identification

After determining the multi-temporal estimation model constructed, datasets for training RF, SVM, and KNN models for maize density are split into 70% for training and 30% for validation, ensuring a balanced assessment of the models’ performance. The results utilized feature recursive elimination to screen various optical and vegetation parameters, thereby enhancing both model performance and interpretability [82].

The model’s estimation accuracy is assessed using R^2^ and RMSE. R^2^ measures the extent to which vegetation parameters explain the variance in maize density. The best possible score is 1, but R^2^ can also be negative, indicating that the model performs worse than a simple mean-based prediction [83]. RMSE measures the square root of the average squared differences between the predicted and actual maize density values, serving as an indicator of the prediction’s average deviation.

(1) $$R^{2}=1-SSR/SST$$

(2) SSR=∑i=1102yi−yi^

(3) $$SST=\sum_{i=1}^{102}\left(y_{i}-y_{i}^{-}\right)$$

(4) $$RMSE=\sqrt{SSR/102}$$

where SSR stands for Sum of Squares Regression, SST represents the Sum of Squares Total, $y_{i}$ denotes the observed value of maize density, yi^ is the predicted value of maize density based on the model, and $y_{i}^{-}$ signifies the mean of the observed values of maize density.

Accuracy evaluation results enable the identification of optimal models for distinct growth stages and datasets, thereby achieving the highest optimal accuracy.

### 4.7. Maize Density Mapping Strategy and Implementation

**Figure 8:**
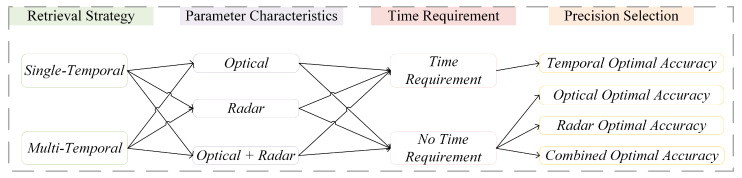
Maize density mapping strategy Map.

In devising a mapping strategy for retrieving maize density over a large area, we have observed that the requirements vary across regions due to differences in data availability and time constraints (Figure 8). In terms of data availability, radar coverage in the study area is not comprehensive and does not cover the entire maize growth cycle. Furthermore, cloud cover represents another challenge in terms of optical data availability. Consequently, it is necessary to determine the optimal mapping strategy based on the available optical and radar imagery in each region. Concerning time constraints, some regions require timely updates on maize density for effective crop monitoring by governmental agencies. In such cases, the primary concern is the promptness of data delivery, with accuracy following as the next priority. Due to the complexity of maize density estimation strategies over large areas, we present the most typical and commonly observed maize mapping results from three typical demonstration counties: Wafangdian in Liaoning Province, Meihekou in Jilin Province, and Qinggang in Heilongjiang Province.

Wafangdian, situated in Liaoning Province, is a preeminent agricultural county and has garnered recognition as a “model county for modern agriculture in China”. The agronomic practice for maize cultivation predominantly employs a sparse planting approach with large-kerneled varieties [40]. Given that Sentinel-1 GRD data does not encompass Wafangdian, and there is an absence of temporal constraints for maize density, the adopted methodology here leverages the zenith of optical feature precision for estimating maize density.

Meihekou, located in Jilin Province, is nestled within one of the globe’s three preeminent “golden maize belts”. Benefiting from the comprehensive coverage of Sentinel-1 GRD and Sentinel-2A datasets and devoid of temporal restrictions for maize density estimation, this region employs an amalgamation of optical and radar features to ascertain maize density with accuracy.

Qinggang, located in Heilongjiang Province, is endowed with an extensive array of optical and radar data and is celebrated as the “maize cradle”. The local administrative bodies are tasked with the prompt evaluation of farmland risk levels and the refinement of crop growth management practices to furnish critical data support for agricultural decision-making and insurance underwriting. The early procurement of maize density metrics is crucial for the strategic enhancement of agricultural productivity [41]. Consequently, a leaf development stage model is employed herein for the precise estimation of maize density.

## 5. Conclusions

This paper developed a method for large-scale and multi-temporal maize density estimation. By incorporating the mapping strategy, this method allows for the mapping of maize density across large-scale areas. It addresses two major challenges in large-scale maize density estimation: First, the multi-temporal model integrates optical and radar to estimate maize density while evaluating the importance of different features at different growth stages. Second, the mapping strategy effectively manages imagery availability and time constraints to map large-scale maize density.

The single-temporal maize density estimation exhibits low accuracy, with difficulties estimated during the tasseling, milk ripening, and maturity stages. The highest accuracy is observed during leaf development (R^2^ = 0.452, RMSE = 0.110). Additionally, we demonstrated the multi-temporal for maize density estimation and recommend using a combination of leaf development, stem elongation, and tasseling to achieve the best accuracy in maize density estimation (R^2^ = 0.602, RMSE = 0.094). In the feature importance ranking, SAVI stands out as the most significant during the tasseling stage, while VV*VH at the stem elongation stage and VV+VH at the leaf development stage also show importance, emphasizing SAVI’s performance during the tasseling stage and the value of cross-polarization. Meanwhile, maize density maps were generated for three typical demonstration counties using the multi-temporal model in conjunction with the mapping strategy.

To our knowledge, this paper represents the first attempt at large-scale maize density estimation and mapping, addressing challenges such as image integration and image availability. By combining the mapping strategy, this multi-temporal model can be easily extended to other regions, providing accurate maize density maps. This advancement has the potential to enhance agricultural resource management and promote the development of precision agriculture.

## Figures and Tables

**Table 1:** The estimation results of single temporal stage maize density.

Grow Stages		Optical		Radar		Optical + Radar		Highest Accuracy	
		R^2^	RMSE	R^2^	RMSE	R^2^	RMSE	R^2^	RMSE
Leaf development (LD)	RF	0.074	0.144	0.303	0.125	**0.452**	**0.110**	**0.452**	**0.110**
	SVR	0.105	0.141	0.179	0.135	0.199	0.116		
	KNN	0.065	0.144	0.096	0.142	0.180	0.135		
Stem elongation (SE)	RF	0.081	0.148	0.298	0.125	**0.408**	**0.115**	**0.408**	**0.115**
	SVR	0.155	0.137	0.221	0.130	0.180	0.109		
	KNN	0.091	0.142	0.149	0.137	0.256	0.129		
Tasseling	RF	0.043	0.145			0.088	0.143	**0.229**	**0.133**
	SVR	0.023	0.147			0.201	0.133		
	KNN	0.091	0.142			**0.229**	**0.133**		
Milk ripening	RF							**0.201**	**0.133**
	SVR			0.031	0.141	**0.201**	**0.133**		
	KNN					0.032	0.147		
Maturity	RF			0.139	0.138			**0.250**	**0.143**
	SVR			**0.250**	**0.143**				
	KNN								

Notes: The null value means R^2^ is negative.

**Table 2:** The estimation results of multi-temporal phases of maize density.

Grow Phases		Optical		Radar		Optical + Radar		Highest Accuracy	
		R^2^	RMSE	R^2^	RMSE	R^2^	RMSE	R^2^	RMSE
Leaf development to Stem elongation	RF	0.125	0.139	0.322	0.123	0.407	0.115		
	SVR	0.061	0.144	0.275	0.127	**0.519**	**0.103**	**0.519**	**0.103**
	KNN	0.137	0.138	0.150	0.137	0.394	0.116		
Leaf development to Tasseling	RF	0.148	0.137	0.332	0.122	**0.602**	**0.094**		
	SVR	0.051	0.145	0.283	0.126	0.567	0.095	**0.602**	**0.094**
	KNN	0.037	0.146	0.201	0.133	0.348	0.121		
Leaf development to Milk ripening	RF	0.131	0.139	0.387	0.116	**0.566**	**0.098**		
	SVR	0.158	0.137	0.276	0.127	0.559	0.095	**0.566**	**0.098**
	KNN	0.171	0.136	0.242	0.130	0.402	0.115		
Leaf development to Maturity	RF	0.276	0.127	0.387	0.116	**0.564**	**0.098**		
	SVR	0.141	0.138	0.276	0.127	0.556	0.099	**0.564**	**0.098**
	KNN	0.243	0.130	0.242	0.130	0.316	0.132		

**Table 3:** Maize density mapping strategy.

Grow Stage/Phase		Optical			Radar			Optical + Radar		
		R^2^	RMSE	MODEL	R^2^	RMSE	MODEL	R^2^	RMSE	MODEL
Single-temporal	Leaf development	0.105	0.141	SVR	0.303	0.125	RF	**0.452**	**0.110**	**RF ^C^**
	Stem elongation	0.155	0.137	SVR	0.298	0.125	RF	0.408	0.115	RF
	Tasseling	0.091	0.142	KNN				0.229	0.133	KNN
	Milk ripening				0.031	0.141	SVR	0.201	0.133	SVR
	Maturity				0.250	0.143	SVR			
Multi-temporal	Leaf development to Stem elongation	0.137	0.138	KNN	0.322	0.123	RF	0.519	0.103	SVR
	Leaf development to Tasseling	0.148	0.137	RF	0.332	0.122	RF	**0.602**	**0.094**	**RF ^B^**
	Leaf development to Milk ripening	0.171	0.136	KNN	0.387	0.116	RF	0.566	0.098	RF
	Leaf development to Maturity	**0.276**	**0.127**	**RF ^A^**	0.387	0.116	RF	0.564	0.098	RF

Notes: The null value means R^2^ is negative. ^A^ represents the mapping strategy for Wafangdian, referred to as W_SM_O_RF, ^B^ represents the mapping strategy for Meihekou, referred to as M_ST_OR_RF, ^C^ represents the mapping strategy for Qinggang, referred to as Q_S_OR_RF. The nomenclature begins with the abbreviation of each county, followed by the abbreviation for the maize growth stage, the data type, and the model used.

**Table 4:** Classification of maize growth stages.

Growth Stage of Maize	Start–End Date
Sowing	5.10–5.30
Leaf development	6.01–6.20
Stem elongation	6.20–7.10
Tasseling	7.10–7.30
Milk ripening	8.01–8.20
Maturity	8.20–9.10

**Table 5:** Vegetation Parameter Characteristics Information.

Index		Formulation	Reference
Optical	NDVI	$\frac{R_{NIR}-R_{RED}}{R_{NIR}+R_{RED}}$	[69]
	SAVI	$\frac{1.5*\left(R_{NIR}-R_{RED}\right)}{R_{NIR}+R_{RED}+0.5}$	[65]
	EVI	$\frac{2.5*\left(R_{NIR}-R_{RED}\right)}{R_{NIR}+6.0*R_{RED}-7.5*R_{BLUE}+1}$	[39]
Radar	VV	$\sigma_{VV}^{0}$	[70]
	VH	$\sigma_{VH}^{0}$	[70]
	VV+VH	$\sigma_{VV}^{0}+\sigma_{VH}^{0}$	[71]
	VV*VH	$\sigma_{VV}^{0}*\sigma_{VH}^{0}$	[72]
	DPSVIm	$\frac{{\sigma_{VV}^{0}}^{2}-\sigma_{VV}^{0}*\sigma_{VH}^{0}}{\sqrt{2}}$	[73]

**Table 6:** Input parameters for maize density estimation during single growth stage.

**Grow Stage**	**Types of Parameters**	**Model Inputs**
Leaf development-Maturity	Optical	NDVI_Growing Stage
		EVI_Growing Stage
		SAVI_Growing Stage
	Radar	VV_Growing Stage
		VH_Growing Stage
		VV+VH_Growing Stage
		VV*VH_Growing Stage
		DPSVIm_Growing Stage
	Optical + Radar	NDVI_Growing Stage
		EVI_Growing Stage
		SAVI_Growing Stage
		VV_Growing Stage
		VH_Growing Stage
		VV+VH_Growing Stage
		VV*VH_Growing Stage
		DPSVIm_Growing Stage

**Table 7:** Input parameters for maize density estimation during multiple growth phases.

**Pronouns**	**Grow Phase Combinations**	**Types of Parameters**
Leaf development to Stem elongation	Leaf development + Stem elongation	Optical
		Radar
		Optical + Radar
Leaf development to Tasseling	Leaf development + Stem elongation + Tasseling	Optical
		Radar
		Optical + Radar
Leaf development to Milk ripening	Leaf development + Stem elongation + Tasseling + Milk ripening	Optical
		Radar
		Optical + Radar
Leaf development to Maturity	Leaf development + Stem elongation + Tasseling + Milk ripening + Maturity	Optical
		Radar
		Optical + Radar

## Data Availability

The original contributions presented in this study are included in the article. Further inquiries can be directed to the corresponding authors.
